# Clinical risk factors associated with rapid renal function decline after nephrectomy

**DOI:** 10.1186/s12885-026-15541-1

**Published:** 2026-01-24

**Authors:** Ying-Che Huang, Yi-Yang Liu, Hui-Ying Liu, Yin-Lun Chang, Hung-Jen Wang, Yen-Ta Chen, Hao-Lun Luo

**Affiliations:** Department of Urology, Kaohsiung Chang Gung Memorial Hospital and Chang Gung University and College of Medicine, No. 123, Dapi Road, Niaosung, Kaohsiung, Taiwan

**Keywords:** Nephrectomy, Estimated glomerular filtration rate, Acute kidney injury, Diabetes mellitus, Risk stratification

## Abstract

**Background:**

Nephrectomy inevitably reduces renal mass and increases the risk of chronic kidney disease (CKD). Identifying predictors of early renal decline after surgery is crucial for optimizing long-term outcomes.

**Methods:**

We retrospectively analyzed 1723 patients with renal cell carcinoma (RCC) or upper tract urothelial carcinoma (UTUC) from the Taiwan Cancer Database linked with the Chang Gung Research Database (2005–2024). Rapid decline was defined as a decrease in estimated glomerular filtration rate (eGFR) > 3 mL/min/1.73 m² between postoperative month 3 and month 15. Postoperative acute kidney injury (AKI) was defined according to KDIGO criteria. Logistic regression was used to evaluate demographic and clinical predictors. Risk stratification was performed according to diabetes mellitus (DM) and AKI.

**Results:**

Of 1723 patients, 720 (41.8%) experienced rapid decline. Compared with those without decline, affected patients were older, more often female, and had higher rates of hypertension, DM, and hyperlipidemia. Preoperative eGFR < 45 and postoperative AKI were also more common. In multivariate analysis, DM (OR 1.56, 95% CI 1.25–1.94, *p* < 0.001) and postoperative AKI (OR 1.85, 95% CI 1.43–2.38, *p* < 0.001) remained independent predictors. Risk stratification showed a stepwise increase in decline: 33.9% without either factor, 47.8% with DM only, 53.3% with AKI only, and 57.8% with both. Patients with both conditions had nearly a 1.7-fold higher risk compared with those without either factor, and Kaplan–Meier analysis confirmed significantly worse outcomes over time (*log-rank p* < 0.05).

**Conclusions:**

DM and postoperative AKI independently predict early renal deterioration after nephrectomy. Patients with both factors represent a high-risk subgroup requiring intensified surveillance and preventive strategies.

**Supplementary Information:**

The online version contains supplementary material available at 10.1186/s12885-026-15541-1.

## Background

Nephrectomy, performed for renal cell carcinoma (RCC) or upper tract urothelial carcinoma (UTUC) [1–3], inevitably results in a reduction of renal mass and increases the risk of subsequent chronic kidney disease (CKD) [4–6]. While many patients maintain stable renal function after surgery, a significant proportion experience early and accelerated decline. Identifying patients at highest risk is essential for postoperative surveillance and timely intervention.

Previous studies have identified several risk factors that increase the likelihood of CKD, including demographic variables such as age and sex, and comorbidities such as hypertension, diabetes mellitus, and cardiovascular disease [7–9]. Perioperative complications, particularly acute kidney injury (AKI), have also been recognized as important contributors to long-term renal deterioration [10]. However, the relative importance of these factors in predicting early renal decline after nephrectomy remains uncertain.

The objective of this study was to evaluate demographic and clinical predictors of early renal function decline following nephrectomy. By systematically analyzing a large, population-based cohort, we aimed to clarify which baseline characteristics and perioperative factors are most strongly associated with early postoperative deterioration in kidney function.

## Methods

### Study population

We identified patients diagnosed with renal cell carcinoma (RCC) or upper tract urothelial carcinoma (UTUC) (ICD codes C64, C65, C66) from the Taiwan Cancer Database (TCDB) linked with the Chang Gung Research Database (CGRD). Patients who underwent nephrectomy between January 1, 2005, and June 30, 2024, were screened. Exclusion criteria included preexisting end-stage renal disease (ESRD), bilateral nephrectomy or nephroureterectomy, no nephrectomy or nephroureterectomy, death within one year after surgery, perioperative dialysis, chemotherapy exposure within one year, missing body mass index (BMI), or absence of follow-up eGFR data. A total of 1723 patients were included in the final analysis.

This study was conducted in accordance with the Declaration of Helsinki and was approved by the Institutional Review Board of Chang Gung Memorial Hospital (IRB Nos. 202401237B0 and 202401236B0). The requirement for informed consent was waived due to the retrospective nature of the study.

### Outcome definitions

The primary outcome was early postoperative renal function decline, defined as an annualized decrease in estimated glomerular filtration rate (eGFR) > 3 mL/min/1.73 m² between postoperative month 3 (baseline) and month 15. This threshold was selected based on prior population-based studies in which an annual eGFR decline of approximately 3 mL/min/1.73 m² represented the upper quartile of renal function loss and substantially exceeded the rate expected from normal aging. In the postoperative setting, this cutoff was considered clinically meaningful for identifying unusually rapid renal deterioration rather than gradual age-related decline [11]. For patients without measurements at exactly these time points, eGFR values were interpolated from adjacent measurements.

Postoperative acute kidney injury (AKI) was defined according to KDIGO criteria as an increase in serum creatinine > 0.3 mg/dL within 48 h, or an increase to > 1.5 times the preoperative baseline within 7 days [12].

### Covariates

Baseline variables included demographic characteristics (age, sex, BMI), comorbidities (hypertension, diabetes mellitus, coronary artery disease, hyperlipidemia, hyperuricemia/gout, renal or ureteral stones, hydronephrosis, polycystic kidney disease, nephrotic syndrome), and preoperative eGFR (≥ 45 vs. <45 mL/min/1.73 m²). Perioperative AKI status was also recorded.

### Statistical analysis

Continuous variables were compared using the Student’s *t*-test, and categorical variables using the chi-square test. Logistic regression models were used to identify predictors of rapid eGFR decline. Variables significant in univariate analysis were included in multivariate models. Risk stratification was performed based on the presence of diabetes mellitus and postoperative AKI. Statistical significance was set at *p* < 0.05.

## Results

### Study cohort

**Fig. 1:**
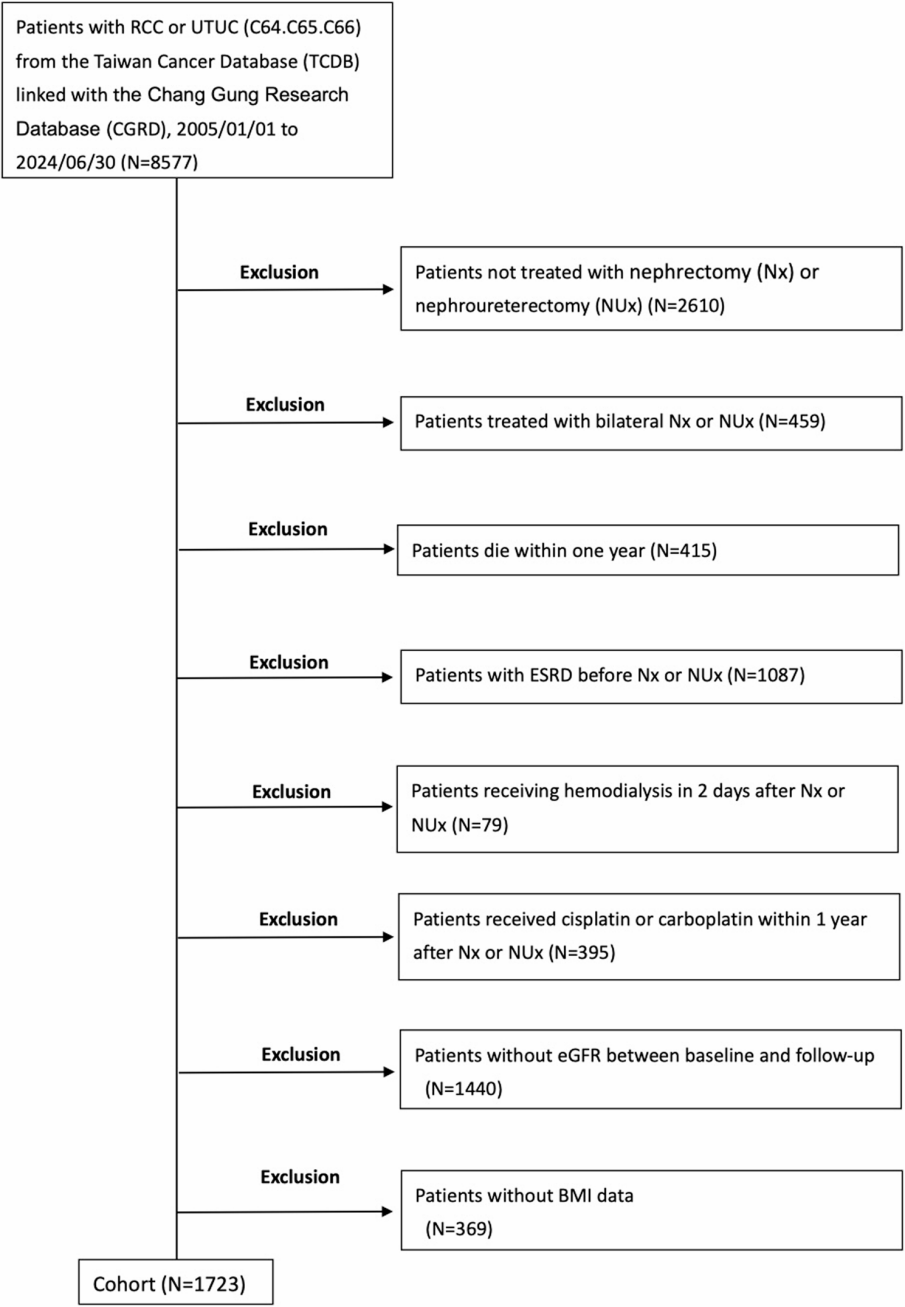
Study cohort flow diagram

A total of 8577 patients with RCC or UTUC were identified from the Taiwan Cancer Database linked with the Chang Gung Research Database between January 1, 2005, and June 30, 2024. After excluding those with preexisting end-stage renal disease, bilateral nephroureterectomy, no nephroureterectomy, death within one year, perioperative dialysis, chemotherapy exposure, missing BMI, or insufficient eGFR data, 1723 patients were included in the final analysis (Fig. 1).

### Patient characteristics

Among the 1723 patients, 720 (41.8%) experienced rapid eGFR decline. Compared with patients without decline, these patients were older (64.5 ± 13.3 vs. 62.8 ± 13.5 years, *p* = 0.011), more frequently female (47.6% vs. 41.4%, *p* = 0.011), and had higher rates of hypertension (68.3% vs. 63.3%, *p* = 0.031), diabetes mellitus (41.3% vs. 29.9%, *p* < 0.001), and hyperlipidemia (32.9% vs. 26.5%, *p* = 0.004). Preoperative eGFR < 45 was more common in the rapid decline group (20.1% vs. 15.3%, *p* = 0.008). Postoperative AKI occurred in 25.0% of rapid decliners versus 14.8% of others (*p* < 0.001) (Table 1).

**Table 1:** Baseline characteristics of patients with and without early postoperative eGFR decline

	Rapid decline (eGFR annual loss of 3 mL/min/1.73 m2)		
	No	Yes	*p*-value
All patients	1003	720	
Age	62.8±13.5	64.5±13.3	*0.011*
Sex (n, %)			
Female	415(41.4%)	343(47.6%)	*0.011*
Male	588(58.6%)	377(52.4%)	
BMI (Mean±SD)	25.3±4.2	25.2±4.2	0.596
HTN (n, %)	635(63.3%)	492(68.3%)	*0.031*
DM (n, %)	300(29.9%)	297(41.3%)	*< 0.001*
CAD (n, %)	165(16.5%)	118(16.4%)	1
Hyperlipidemia (n, %)	266(26.5%)	237(32.9%)	*0.004*
Hyperuricemia/ Gout (n, %)	29(2.9%)	13(1.8%)	0.15
Renal and ureteral stones (n, %)	72(7.2%)	54(7.5%)	0.8
Hydronephrosis (n, %)	127(12.7%)	107(14.9%)	0.189
Polycystic kidney disease (n, %)	1(0.1%)	2(0.3%)	0.575
Nephrotic syndrome (n, %)	8(0.8%)	3(0.4%)	0.377
Preoperative eGFR			*0.008*
G1-3a ( > = 45)	850(84.8%)	575(79.9%)	
G3b-5 (< 45)	153(15.3%)	145(20.1%)	
Postoperative AKI (Yes, No)	148(14.8%)	180(25%)	*< 0.001*
Follow up Months (Mean±SD)	59.7±36.5	61.9±36.5	0.228

### Regression analysis

Univariate analysis identified older age, female sex, hypertension, diabetes mellitus, hyperlipidemia, preoperative eGFR < 45, and postoperative AKI as significant risk factors. In multivariate models, only diabetes (OR 1.56, 95% CI 1.25–1.94, *p* < 0.001) and postoperative AKI (OR 1.85, 95% CI 1.43–2.38, *p* < 0.001) remained independent predictors (Table 2).

**Table 2:** Univariate and multivariate logistic regression analysis of clinical factors associated with early postoperative eGFR decline

Variable	Univariate		Multivariate	
	OR (95%CI)	*p*-value	OR (95%CI)	*p*-value
Age	1.01 (1-1.02)	0.012	1 (0.99–1.01)	0.578
Male	0.78 (0.64–0.94)	0.01	0.83 (0.68–1.01)	0.067
HTN	1.25 (1.02–1.53)	0.031	1.04 (0.83–1.31)	0.711
DM	1.65 (1.35–2.01)	< 0.001	1.56 (1.25–1.94)	< 0.001
Hyperlipidemia	1.36 (1.1–1.68)	0.004	1.14 (0.91–1.43)	0.26
Preoperative eGFR < 45	1.4 (1.09–1.8)	0.008	1.03 (0.78–1.36)	0.838
Postoperative AKI	1.93 (1.51–2.46)	< 0.001	1.85 (1.43–2.38)	< 0.001

### Risk stratification

The combined effect of diabetes and AKI showed a stepwise increase in the incidence of rapid decline. As summarized in Table 3, the rates of rapid decline were 33.9% in patients without either factor (Group 1), 47.8% in those with diabetes only (Group 2), 53.3% in those with AKI only (Group 3), and 57.8% in those with both diabetes and AKI (Group 4). Patients with both conditions had approximately a 1.7-fold higher risk compared with the lowest-risk group.

**Table 3:** Incidence of rapid eGFR decline stratified by diabetes mellitus (DM) and postoperative acute kidney injury (AKI)

Group	DM	postoperative AKI	Number of Patients	Rapid eGFR Decline (%)
Group 1	No	No	914	33.9%
Group 2	Yes	No	481	47.8%
Group 3	No	Yes	212	53.3%
Group 4	Yes	Yes	116	57.8%

**Fig. 2:**
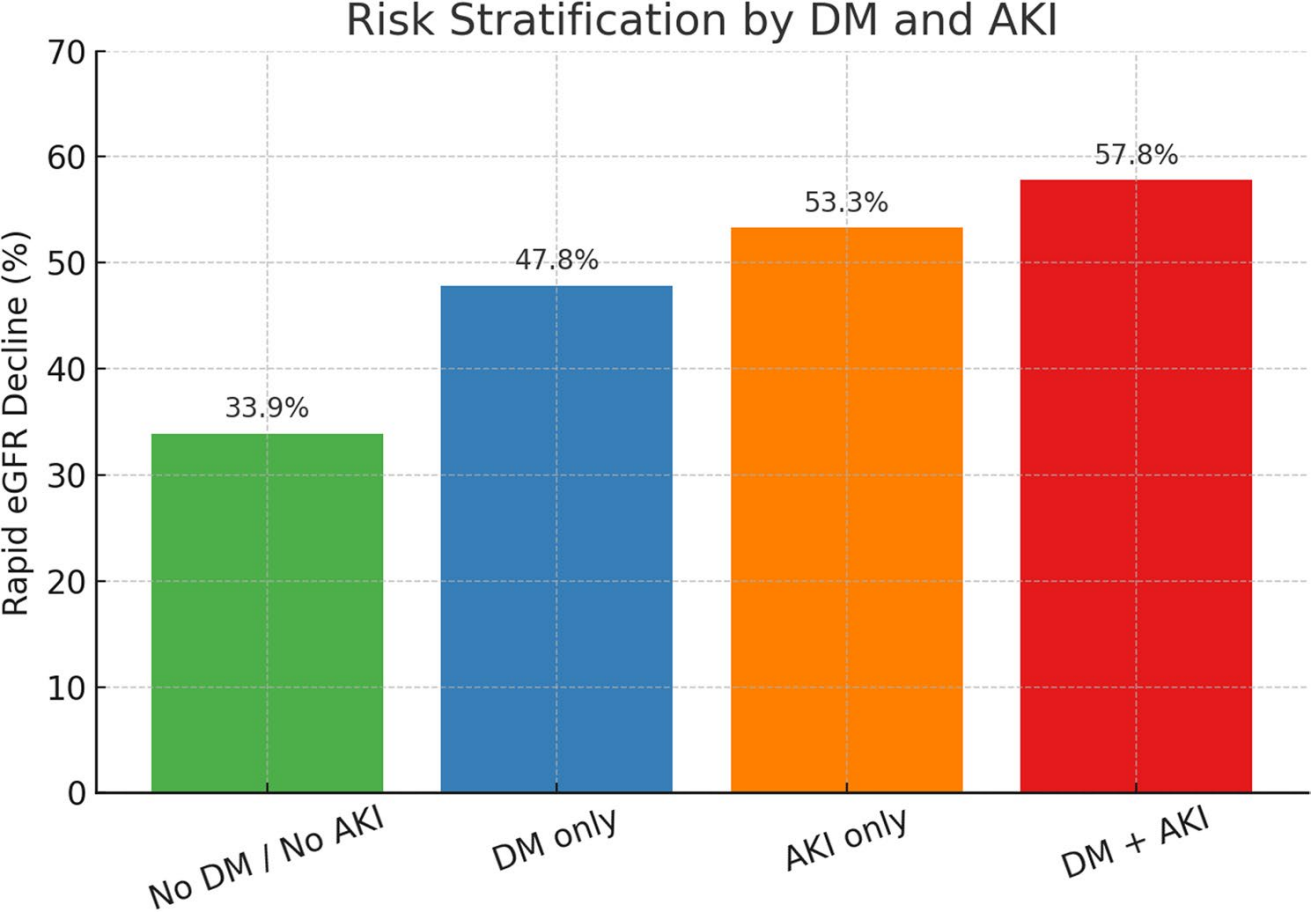
Patients were stratified into four groups according to the presence of diabetes mellitus (DM) and postoperative acute kidney injury (AKI)

**Fig. 3:**
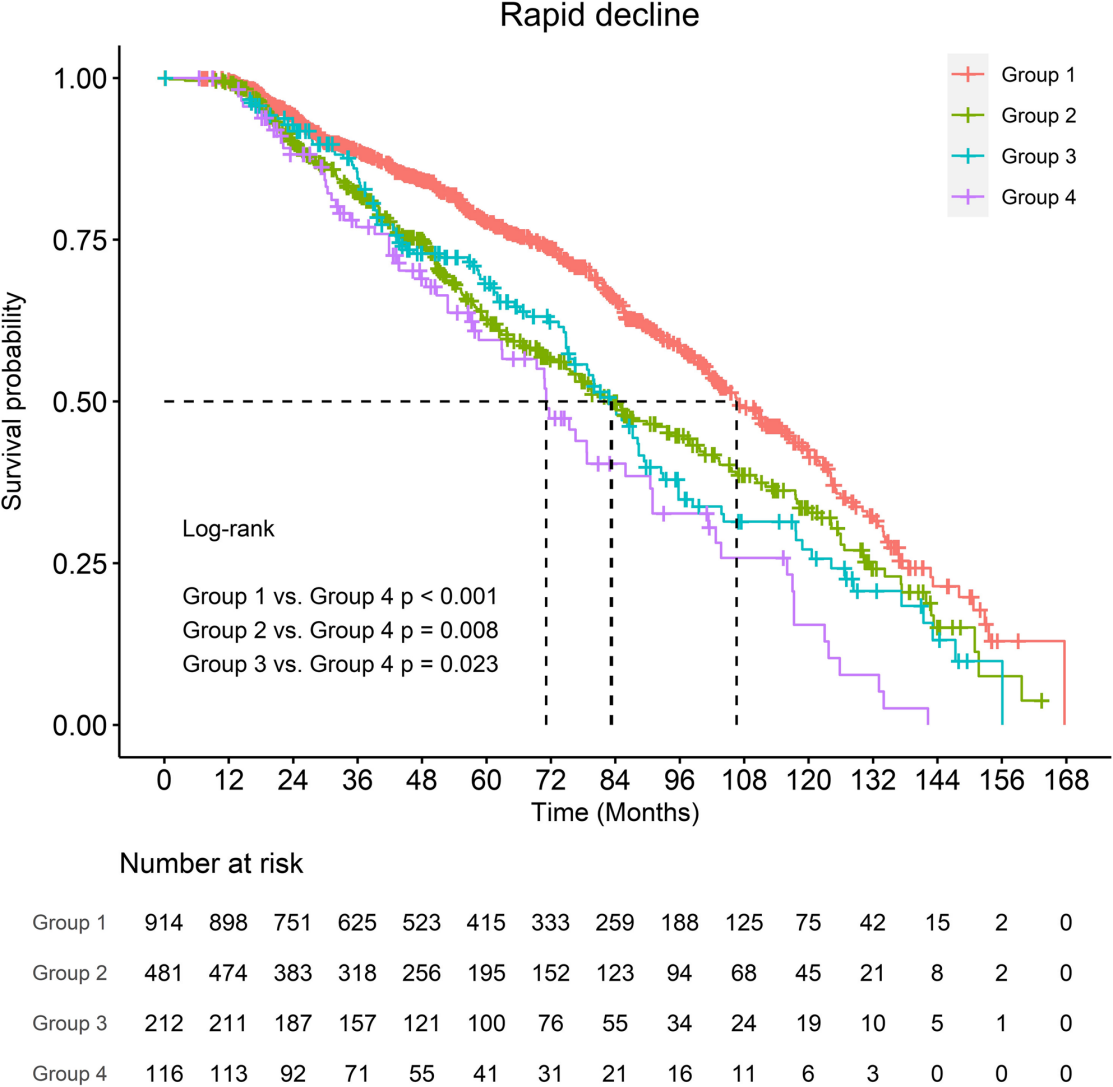
Kaplan–Meier analysis of early postoperative eGFR decline stratified by diabetes mellitus (DM) and postoperative acute kidney injury (AKI)

To provide visual context, Fig. 2 displays the distribution of rapid decline across the four groups, and Fig. 3 demonstrates the Kaplan–Meier curves. Patients in Group 4 showed the steepest deterioration in renal function, whereas Group 1 had the most favorable trajectory. The log-rank test confirmed significant differences between Group 4 and the other groups (*p* < 0.05).

### Baseline characteristics according to postoperative AKI status

Patients who developed postoperative AKI were older, more likely to be female, had lower preoperative eGFR, lower body mass index, and a higher prevalence of hyperlipidemia and hydronephrosis compared with those without AKI (Supplementary Table S1).

## Discussion

In this large cohort study, more than 40% of patients experienced early postoperative eGFR decline after nephrectomy. Importantly, diabetes mellitus and postoperative AKI were identified as the strongest independent predictors, whereas preoperative CKD was not independently associated after adjustment. These findings suggest that perioperative events and metabolic comorbidities, rather than baseline renal status alone, are the key drivers of early deterioration.

Using an annual eGFR decline > 3 mL/min/1.73 m² allowed us to focus on clinically meaningful early postoperative renal deterioration rather than gradual age-related decline.

Although nephroureterectomy (NUx) and nephrectomy differ in surgical field, the primary determinant of postoperative renal function in both procedures is the loss of functional renal parenchyma. NUx inherently involves removal of the entire kidney, and the additional distal ureter and bladder cuff excision does not directly affect glomerular filtration. Previous studies have consistently demonstrated that postoperative AKI is a potent risk factor for chronic kidney disease (CKD) progression. As early as 2011, large cohort studies showed that patients who developed AKI after surgery had a significantly higher likelihood of long-term renal dysfunction compared with those without AKI [13]. More recent investigations across major surgical populations have confirmed the strong association between AKI and both short-term morbidity and long-term CKD risk [14, 15]. Mechanistically, AKI and CKD share overlapping pathways, including tubular injury, interstitial fibrosis, and maladaptive repair. Even in the absence of preexisting CKD, AKI can trigger irreversible damage that ultimately results in progressive deterioration of renal function [16].

Our findings extend these observations to patients undergoing nephrectomy, a population already predisposed to renal function loss due to nephron reduction. We demonstrated that postoperative AKI independently predicted rapid eGFR decline, underscoring the importance of perioperative renal protection. Notably, preoperative CKD (eGFR < 45) was not independently associated with outcomes after adjustment, suggesting that the occurrence of AKI and the presence of diabetes, rather than baseline CKD status, are the principal determinants of early deterioration.

The baseline differences observed between patients with and without postoperative AKI likely reflect pre-existing renal and metabolic vulnerability rather than confounding, as AKI was modeled as an explanatory variable rather than the primary outcome. Clinical factors such as older age, female sex, lower preoperative eGFR, lower BMI, hyperlipidemia, and hydronephrosis may predispose patients to the development of AKI through reduced renal reserve, altered metabolic status, or impaired urinary drainage.

However, these factors were not independently associated with rapid postoperative eGFR decline in the primary outcome analysis. This suggests that susceptibility to AKI does not necessarily translate into accelerated postoperative renal function loss, and that postoperative AKI itself—rather than its predisposing conditions—plays a more critical role in determining early renal function trajectories after nephrectomy.

Notably, preoperative CKD defined as eGFR < 45 mL/min/1.73 m² did not remain an independent predictor of rapid postoperative renal decline after multivariable adjustment. This finding differs from some prior reports [17, 18] and warrants further consideration. One possible explanation is that the impact of postoperative AKI outweighed that of baseline renal function. AKI is a strong determinant of long-term renal outcomes and may dominate multivariable models, thereby attenuating the statistical effect of preoperative eGFR. In addition, patients with end-stage renal disease were excluded from this cohort, which reduced variability in the lower range of baseline renal function and may have limited its independent contribution.

Finally, while our outcome focused on postoperative eGFR decline, emerging evidence suggests that preoperative eGFR trajectories may provide complementary information beyond a single baseline measurement in reflecting renal vulnerability [19, 20]. Unfortunately, longitudinal preoperative eGFR data were not available in this dataset. Together, these factors may explain why baseline eGFR alone was not independently associated with early postoperative renal deterioration in our analysis.

The adverse role of diabetes in postoperative renal outcomes has also been well documented. Clinical studies have reported that diabetes impairs renal recovery after nephrectomy and increases the risk of AKI across diverse surgical settings [21–23]. Experimental evidence further indicates that diabetic kidneys are more vulnerable to ischemia–reperfusion injury, characterized by increased tubular apoptosis and early interstitial fibrotic responses mediated by hyperglycemia-associated profibrotic signaling. Under hyperglycemic conditions, activation of the JAK–STAT pathway enhances transforming growth factor-β (TGF-β) expression, which promotes tubulointerstitial fibrotic remodeling through dysregulated extracellular matrix turnover, thereby impairing renal repair and functional recovery after injury [24–27].

In addition to profibrotic responses, diabetic kidneys exhibit impaired repair capacity compared with non-diabetic kidneys [28]. In the diabetic milieu, hyperglycemia promotes the accumulation of advanced glycation end products (AGEs) and activation of the receptor for advanced glycation end products (RAGE) signaling, leading to oxidative stress and reduced tubular transport efficiency. These metabolic disturbances induce mitochondrial stress and establish a pro-inflammatory tubular environment, which may predispose the kidney to impaired recovery following surgical insult [26, 29].

When AKI develops, these pre-existing vulnerabilities are further amplified by inflammatory cascades involving both resident and infiltrating immune cells [30, 31]. In addition to immune cells, injured tubular epithelial cells actively participate in sustaining local inflammation through cytokine and chemokine signaling [26]. Rather than reflecting gradual chronic kidney disease progression, these early maladaptive repair responses may interfere with compensatory adaptation following nephron loss, thereby contributing to an accelerated decline in renal function.

Consequently, diabetes not only predisposes patients to AKI but also amplifies its downstream functional consequences, creating a compounded “double-hit” effect. These mechanisms are consistent with our observation that patients with both diabetes and AKI had nearly a 60% risk of rapid renal decline, the highest among all subgroups.

Clinically, these findings highlight two key implications. First, patients with diabetes require optimized perioperative management, including strict glycemic control, careful fluid balance, and avoidance of nephrotoxic agents. Second, preventing postoperative AKI should be a priority. Strategies such as maintaining adequate hydration, minimizing ischemia time, and monitoring renal function closely in the early postoperative period may reduce the risk of renal deterioration. Together, these measures can help protect renal function in high-risk patients.

The strengths of this study include its large cohort size, long-term follow-up, and the use of standardized KDIGO criteria for defining postoperative acute kidney injury, along with a literature-supported definition of rapid renal function decline based on annualized eGFR change. The linkage of the TCDB with a comprehensive hospital-based dataset provided robust and reliable information, minimizing misclassification and enhancing generalizability within the local population. By applying a consistent definition of rapid postoperative renal function decline and multivariate regression analyses, we were able to disentangle the relative contributions of baseline comorbidities and perioperative events.

Nevertheless, several limitations should be acknowledged. First, the retrospective design may introduce confounding and selection bias. Second, several granular perioperative variables—including surgical approach (open, laparoscopic, or robotic), ischemia time, operative duration, intraoperative hypotension, blood loss, transfusion, and tumor location—were not comprehensively available in the current TCDB–CGRD linkage and therefore could not be reliably incorporated into multivariable analyses. These factors may individually influence postoperative renal outcomes and could not be evaluated separately in the present study. To partially account for perioperative influences, we incorporated postoperative AKI as a representative perioperative indicator, as AKI reflects the cumulative impact of intraoperative and early postoperative insults, including ischemia–reperfusion injury, hemodynamic instability, surgical complexity, and nephron mass loss. Nevertheless, we acknowledge that AKI cannot fully substitute for detailed perioperative metrics. Inclusion of more granular surgical and hemodynamic data may provide additional insight and should be addressed in future prospective studies. Third, although modern systemic therapies may influence renal function, the use of adjuvant immunotherapy and targeted therapy after curative surgery remained limited during the majority of the study period in the TCDB population. In addition, patients with advanced disease or early mortality were excluded, further reducing the likelihood that intensive postoperative systemic therapy substantially influenced renal outcomes. Detailed information regarding treatment timing and dosing was not comprehensively available in the linked dataset, precluding reliable adjustment in the analysis. Nevertheless, it is worth noting that commonly used targeted agents and immune checkpoint inhibitors in RCC and UTUC are generally considered feasible in patients with impaired renal function and are not known to exert major direct nephrotoxic effects or to rely heavily on renal clearance. Therefore, although residual confounding cannot be entirely excluded, the overall impact of postoperative systemic therapies on early eGFR trajectories in this cohort is likely to be limited. Finally, the study was conducted within a single healthcare system in Taiwan, which may limit generalizability to other populations.

Future prospective studies with more granular perioperative data and validation in diverse populations are warranted to confirm these findings and to develop targeted strategies for high-risk patients.

## Conclusions

Early postoperative renal function decline occurred in over 40% of patients after nephrectomy. Diabetes mellitus and postoperative AKI were the strongest independent predictors, while preoperative CKD was not independently associated with deterioration after adjustment. Patients with both diabetes and AKI had nearly a 60% risk of decline, underscoring the compounded adverse effect of these conditions.

These findings highlight the need for optimized perioperative management in diabetic patients and the implementation of strategies to prevent postoperative AKI in order to preserve renal function and improve long-term outcomes following nephrectomy.

## Supplementary Information

Supplementary Material 1: Supplementary Table S1. Baseline characteristics of patients with and without postoperative acute kidney injury (AKI).

## Data Availability

The data that support the findings of this study are available from Chang Gung Memorial Hospital but restrictions apply to the availability of these data, which were used under license for the current study, and so are not publicly available. Data are however available from the authors upon reasonable request and with permission of Chang Gung Memorial Hospital.

## Abbreviations

TCDB: The Taiwan Cancer Database
CGRD: The Chang Gung Research Database
AKI: Acute Kidney Injury
BMI: Body Mass Index
CAD: Coronary Artery Disease
CKD: Chronic Kidney Disease
DM: Diabetes Mellitus
eGFR: Estimated Glomerular Filtration Rate
ESRD: End-Stage Renal Disease
HTN: Hypertension
RCC: Renal Cell Carcinoma
UTUC: Upper Tract Urothelial Carcinoma
