# Supplementary material for: Clinical risk factors associated with rapid renal function decline after nephrectomy
Source: BMC Cancer. 2026 Jan 24;26:268. doi: 10.1186/s12885-026-15541-1 (PMC12917967; doi:10.1186/s12885-026-15541-1)
Supplement: Supplementary file 1 — Supplementary Material 1: Supplementary Table S1. Baseline characteristics of patients with and without postoperative acute kidney injury (AKI). [file 12885_2026_15541_MOESM1_ESM.docx]

Supplementary Table S1. Baseline characteristics of patients with and without postoperative acute kidney injury (AKI)

|  | Postoperative AKI | |  |
| --- | --- | --- | --- |
|  | No | Yes | p-value |
| All patients | 1395 | 328 |  |
| Age | 62.9±13.4 | 66±13.3 | *<0.001* |
| Sex (n, %)  Female  Male | 575 (41.2%)  820 (58.8%) | 183 (55.8%)  145 (44.2%) | *<0.001* |
| BMI (Mean±SD) | 25.4±4.2 | 24.6±4 | 0.005 |
| HTN (n, %) | 906 (65%) | 221 (67.4%) | *0.405* |
| DM (n, %) | 481 (34.5%) | 116 (35.4%) | *0.762* |
| CAD (n, %) | 230 (16.5%) | 53 (16.2%) | 0.885 |
| Hyperlipidemia (n, %) | 392 (28.1%) | 111 (33.8%) | *0.04* |
| Hyperuricemia/ Gout (n, %) | 31 (2.2%) | 11 (3.4%) | 0.232 |
| Renal and ureteral stones (n, %) | 106 (7.6%) | 20 (6.1%) | 0.348 |
| Hydronephrosis (n, %) | 155 (11.1%) | 79 (24.1%) | <0.001 |
| Polycystic kidney disease (n, %) | 2 (0.1%) | 1 (0.3%) | 0.528 |
| Nephrotic syndrome (n, %) | 10 (0.7%) | 1 (0.3%) | 0.399 |
| Preoperative eGFR  G1-3a (>=45)  G3b-5 (<45) | 1220 (87.5%)  175 (12.5%) | 205 (62.5%)  123 (37.5%) | *<0.001* |
